# Letter to the Editor regarding “Anatomic Total Shoulder Arthroplasty Outcomes Were Not Negatively Affected by the COVID-19 Pandemic”

**DOI:** 10.1055/s-0045-1806815

**Published:** 2025-06-14

**Authors:** Hinpetch Daungsupawong, Viroj Wiwanitkit

**Affiliations:** 1 Private academic consultant, Phonhong, Vientiane, Laos.; 2 Saveetha Institute of Medical and Technical Sciences, Saveetha University, Chennai, Tamil Nadu, India.

Dear Editor,

The article “Anatomic Total Shoulder Arthroplasty Outcomes Were Not Negatively Affected by the COVID-19 Pandemic” 1 is the topic of discussion in this letter. In this study, the range of motion, strength, and results of physical therapy (PT) were compared in patients submitted to reverse total shoulder arthroplasty (RTSA) in 2019 and 2020. The findings demonstrated that forward elevation, external rotation, and internal rotation significantly improved in both groups. In contrast to the 2019 group, patients in 2020 underwent PT for a shorter period and completed fewer sessions. In 2020, however, at the last follow-up, patients reported a low mean pain score.

The comparatively small sample size of this study—24 patients in 2019 and 27 in 2020—is one of its shortcomings. This could induce bias and limit the application of the results. Furthermore, recall bias or the report of inaccuracies may result from the data collection strategy, namely due to the dependence on patient-reported measures during phone calls made in 2022.

In the future, it would be beneficial to carry out a bigger, randomized controlled trial to learn more about the variations in results among patients submitted to RTSA at different times. Furthermore, longitudinal research could be performed to evaluate the long-term impacts of PT duration and completion on patient outcomes. Future studies could investigate the causes of the decline in the number of completed PT sessions and their duration in 2020, as well as any effects this may have had on functional results and overall patient satisfaction. All things considered, this study offers insightful information on the results of RTSA patients and emphasizes how crucial it is to track PT length and completion to enhance outcomes.
